# Nutmeg ( *Myristica fragrans* Houtt.) essential oil: A review on its composition, biological, and pharmacological activities

**DOI:** 10.1002/ptr.7491

**Published:** 2022-05-13

**Authors:** Kaliyaperumal Ashokkumar, Jesus Simal‐Gandara, Muthusamy Murugan, Mannananil Krishnankutty Dhanya, Arjun Pandian

**Affiliations:** ^1^ Cardamom Research Station Kerala Agricultural University Idukki Kerala India; ^2^ Nutrition and Bromatology Group, Department of Analytical Chemistry and Food Science, Faculty of Science Universidade de Vigo Ourense Spain; ^3^ Department of Biotechnology PRIST Deemed University Thanjavur Tamil Nadu India

**Keywords:** biological activities, chemical composition, essential oil, *Myristica fragrans*, myristicin, nutmeg, sabinene

## Abstract

*Myristica fragrans* (Houtt.) is an evergreen tree native to the Maluku Islands, Indonesia. *M*. *fragrans* kernel is extensively used in Indian traditional medicines to treat various diseases. Several studies attempt to compile and interpret the pharmacological potential of *Myristica fragrans* (Houtt.) aqueous and various chemical extracts. Thus, the pharmacological potential of nutmeg essential oil has not been reviewed phytochemically and pharmacologically. Therefore, the present study aimed to share appropriate literature evidence regarding the plant essential oil chemical composition and therapeutic potential of *Myristica fragrans* essential oil (MFEO). MFEO of leaf, mace, kernel, and seed were used worldwide as potential Ayurvedic medicine and fragrance. MFEO extracted by various methods and oil yield was 0.7–3.2, 8.1–10.3, 0.3–12.5, and 6.2–7.6% in leaf, mace, seed, and kernel. The primary chemical constituents of MFEO were sabinene, eugenol, myristicin, caryophyllene, β‐myrcene, and α‐pinene. Clinical and experimental investigations have confirmed the antioxidant, antimicrobial, antiinflammatory, anticancer, antimalarial, anticonvulsant, hepatoprotective, antiparasitic, insecticidal, and nematocidal activities of MFEO. It is the first attempt to compile oil yield, composition, and the biological activities of MFEO. In future, several scientific investigations are required to understand the mechanism of action of MFEO and their bioactive constituents.

## 1 INTRODUCTION

Nutmeg (*Myristica fragrans* Houtt.) belongs to the Myristicaceae family. The plant is native to the Maluku Islands of Indonesia; however, it is extensively distributed to Grenada, India, Sri Lanka, Mauritius, South Africa, and the USA (Francis, James, Varughese, & Nair, 2019). The nutmeg seed has outer, red arils called mace and an inner, brown kernel called nutmeg, both of which are used as a spice (Abourashed & El‐Alfy, 2016; Periasamy, Karim, Gibrelibanos, Gebremedhin, & Gilani, 2016). In traditional medicine, different parts of the plant mentioned traditionally are used to cure various diseases. However, in Indian Ayurvedic medicine, nutmeg has been used to treat anxiety, nausea, diarrhea, cholera, stomach cramps, parasites, paralysis, and rheumatism and is also used as an aphrodisiac (Gils & Cox, 1994; Ziyatdinova, Ziganshina, Cong, & Budnikov, 2016). Furthermore, in Pakistan traditional medicine, the nutmeg plant has been used to treat hypertension (Malik et al., 2018).

The essential oil (EO) of *M*. *fragrans* is a colorless‐to‐light yellow liquid with a distinct spicy odor (Nikolic et al., 2021). Several types of research on the plant's EO have been conducted in various parts of the world (Ashokkumar, Vellaikumar, Murugan, Dhanya, & Aiswarya, 2022; Atta‐ur‐Rahman et al., 2000; Dupuy et al., 2013; Ogunwande, Olawore, Adeleke, & Ekundayo, 2003). Hydrodistillation, steam distillation, supercritical fluid extraction, microwave, and ultrasound‐assisted techniques were used to extract the MFEO (Ashokkumar et al., 2022; Azwanida, 2015). The oil yield of nutmeg kernels ranged from 5 to 15% (Barceloux, 2009). MFEO has chiefly monoterpenes (sabinene, β‐pinene, β‐terpineol, p‐menth‐8‐en‐1‐ol, and terpinen‐4‐ol), phenylpropene (eugenol, methyl eugenol, and myristicin), sesquiterpenes (germacrene D and β‐bergamotene), and other constituents (Atta‐ur‐Rahman et al., 2000; Dupuy et al., 2013; Francis et al., 2019). The major constituents of leaf were sabinene, eugenol, myristicin, caryophyllene, and β‐myrcene. Sabinene, α‐pinene, β‐pinene, d‐limonene, and 3‐carene were predominant constituents of mace. The major constituents of the kernel and seed were sabinene, α‐pinene, β‐pinene, d‐limonene, and β‐myrcene (Ashokkumar et al., 2022).

Several scientific reports say that MFEO has potential antioxidant, antimicrobial, antiinflammatory, antiulcer, anticancer, aphrodisiac, and various other activities (Das et al., 2020; Hiranrat & Hiranrat, 2019; Kholibrina & Aswandi, 2021; Nikolic et al., 2021; Özkan et al., 2018; Purkait, Bhattacharya, Bag, & Chattopadhyay, 2018; Thileepan, Thevanesam, & Kathirgamanathar, 2018; Valente, Jham, Jardim, Dhingra, & Ghiviriga, 2015). The pharmacological potential of *M*. *fragrans* crude extracts/various organic chemical extracts was reviewed and published by several researchers, but the pharmacological activities of MFEO and their active constituents were not yet compiled. Therefore, the present study aimed to provide an overview and critically analyze the reported botanical description, essential oil yield, chemical composition of MFEO, and its biological and pharmacological potentials conferring to published literature up to March 2022 and identify the remaining gaps for further investigations. Furthermore, the review seeks to take peoples and researchers attention to the wide‐ranging biological features of MFEO and its active ingredients to improve their use in the future.

## 2 MATERIALS AND METHODS

A comprehensive review of the literature on *M*. *fragrans* EO yield, chemical composition, pharmacology, and biological studies was conducted, with data compiled using a variety of search engines and publishing houses, including Science Direct, Springer, PubMed, Google Scholar, Taylor and Francis, Frontiers, and NCBI. Other literature sources, such as Wikipedia, ethnobotanical publications, and various online domains, were also examined to obtain as much information about *M*. *fragrans* as possible. *Myristica fragrans*, nutmeg, mace, therapeutic uses of *Myristica fragrans* essential oil, phytochemicals of *Myristica fragrans* essential oil, pharmacological activities of *Myristica fragrans* essential oil, botany of *Myristica fragrans*, and numerous synonyms were also utilized in the literature search.

## 3 BOTANICAL DESCRIPTION

**FIGURE 1:**
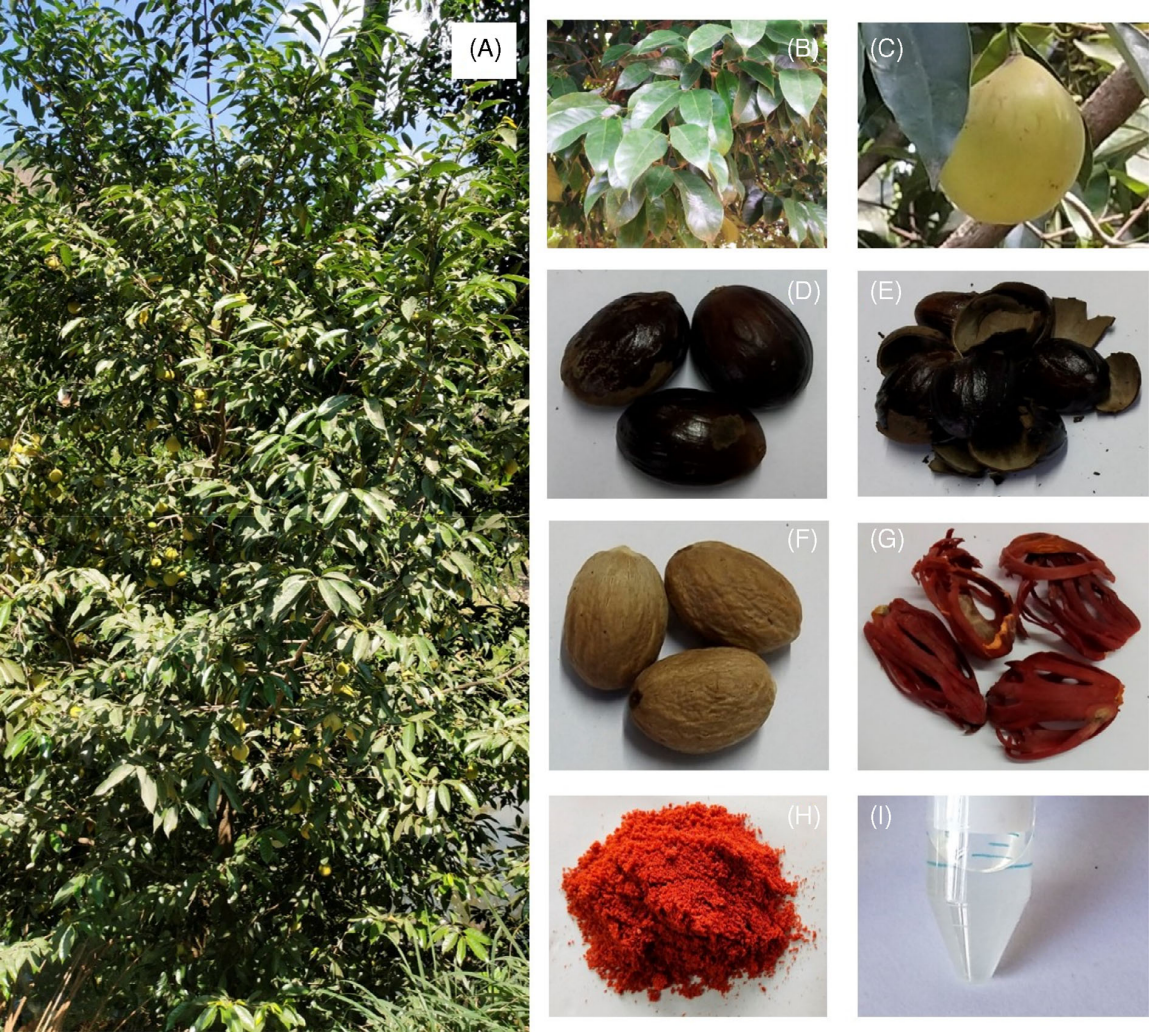
Morphological identification of *Myristica fragrans* Houtt. (a) The young tree; (b) leaves of the plant; (c) fruit; (d) seed; (e) rind; (f) kernel (nutmeg); (g) mace (aril); (h) ground powder of mace; and (i) mace essential oil

*Myristica fragrans* is an evergreen tree, grown up to 20–25 ft high with greyish brown soft bark and spreading branches (Periasamy et al., 2016). The plant is grown well in warm, humid climate, with an elevation of 1,000 m above sea level with 150–250 cm rainfall. The plant has been reported to have four chromosome numbers such as 2*n* = 38 (Dhamayanthi & Krishnamoorthy, 1999), 2*n* = 41 (Nair, 2019), 2*n* = 42 (Purseglove et al., 1981), and 2*n* = 44 (Nair, 2019). However, chromosome number 2*n* = 44 is predominant at the nutmeg seedling stage (Nair, 2019). The leaves are aromatic, dark green, glossy above, alternate, oblong, glabrous, and acuminate (Periasamy et al., 2016). Flowers are usually dioecious, occasionally monoecious with variable sex expression, small axillary, sub‐umbellate racemes, compound, or sometimes forked (Haldankar et al., 2007). The pedicels and peduncles have a glabrous appearance. The fruit has a fleshy pericarp and is spherical; the fruit skin is yellowish and splits into two longitudinal valves. The mace (aril) is a fleshy scarlet aril that is laciniate, folded, and envelops the nut when wet; when dry, it is considerably hornier, yellowish‐brown in color, and highly brittle (Wallis, 1985). The nut is oval or broadly ovate, with a hard, rough, dark‐brown, glossy shell that is pale and smooth on the inside and is approximately half a line thick. When young, the seed/kernel is oval, pale brown, and soft, but it quickly shrivels and has irregular, vertical lines or furrows on its surface and rich in oil (Naeem, Rehman, Mushtaq, & Ghania, 2016; Periasamy et al., 2016). The tree bears fruit all year, but the best time to harvest is between April and November. The morphological identification of whole plant, leaves, fruit, seed, rind, kernel, and mace (aril) of *M*. *fragrans* is shown in Figure 1.

### 3.1 MFEO yield

The essential oil yield of *M*. *fragrans* leaves, mace, seed, and kernel were 0.7–3.2, 8.1–10.3, 0.3–12.5, and 6.2–7.6%, respectively. The variation in EO depends upon the source and extraction method (Table 1). MFEO was extracted by various methods including, hydrodistillation (Ashokkumar et al., 2022; Carolina & Maman, 2016; Ibrahim et al., 2020; Mickus et al., 2021; Muchtaridi et al., 2010; Nikolic et al., 2021; Waman, 2020), steam distillation (Al‐Jumaily & Al‐Amiry, 2012; Carolina & Maman, 2016; Purseglove et al., 1981), microwave‐assisted hydrodistillation (Sagarika et al., 2018), and CO_2_ supercritical fluid extraction (Hanif et al., 2010). Ashokkumar et al. (2022) found that the Western Ghats, southern India, grown nutmeg leaf EO yield (3.2%) was greater than that found in Bogor, West Java, Indonesia, grown nutmeg leaf (0.7%; Carolina & Maman, 2016). Furthermore, the yields of nutmeg seed oil grown in South India (5.2%) were lower than those grown in Brazil (7.1%; Valente et al., 2015). The differences in EO yield of nutmeg might be due to changes in soil type, location, origin, extraction methods, and environmental conditions (Ashokkumar et al., 2022).

**TABLE 1:** Yield of essential oil from various parts of *Myristica fragrans*

Parts	Extraction method	Volatile oil (%)	References
Leaf	Hydrodistillation	3.2	Ashokkumar et al. (2022)
Leaf	Steam distillation	0.7	Carolina and Maman (2016)
Mace	Hydrodistillation	8.1	Ashokkumar et al. (2022)
Mace	Hydrodistillation	10.3	Muhammad et al. (2016)
Kernel	Hydrodistillation	6.2	Ashokkumar et al. (2022)
Kernel	Hydrodistillation	7.6	Muhammad et al. (2016)
Seed	Hydrodistillation	5.2	Ashokkumar et al. (2022)
Seed	Hydrodistillation	5.1–7.2	Waman (2020)
Seed	Hydrodistillation	6.9	Muchtaridi, Subarnas, Apriyantono, and Mustarichie (2010)
Seed	Hydrodistillation	5.8	Ibrahim, Cantrell, Jeliazkova, Astatkie, and Zheljazkov (2020)
Seed	Hydrodistillation	7.1	Nikolic et al. (2021)
Seed	Hydrodistillation	8.4	Mickus et al. (2021)
Seed	Steam distillation	4.5–7.5	Al‐Jumaily and Al‐Amiry (2012)
Seed	Steam distillation	0.3	Carolina and Maman (2016)
Seed	Steam distillation	12.5	Purseglove, Brown, Green, and Robbins (1981)
Seed	Microwave‐assisted hydrodistillation	3.8–5.8	Sagarika et al. (2018)
Kernel	CO_2_ supercritical fluid extraction	5.9	Hanif et al. (2010)

Among the extraction methods, classical hydrodistillation by Clevenger apparatus is a widely used method of volatile oil estimation than other techniques. Owing to the prominence of nutmeg essential oil yield have been reviewed and summarized in the Table 1.

### 3.2 MFEO chemical composition

**FIGURE 2:**
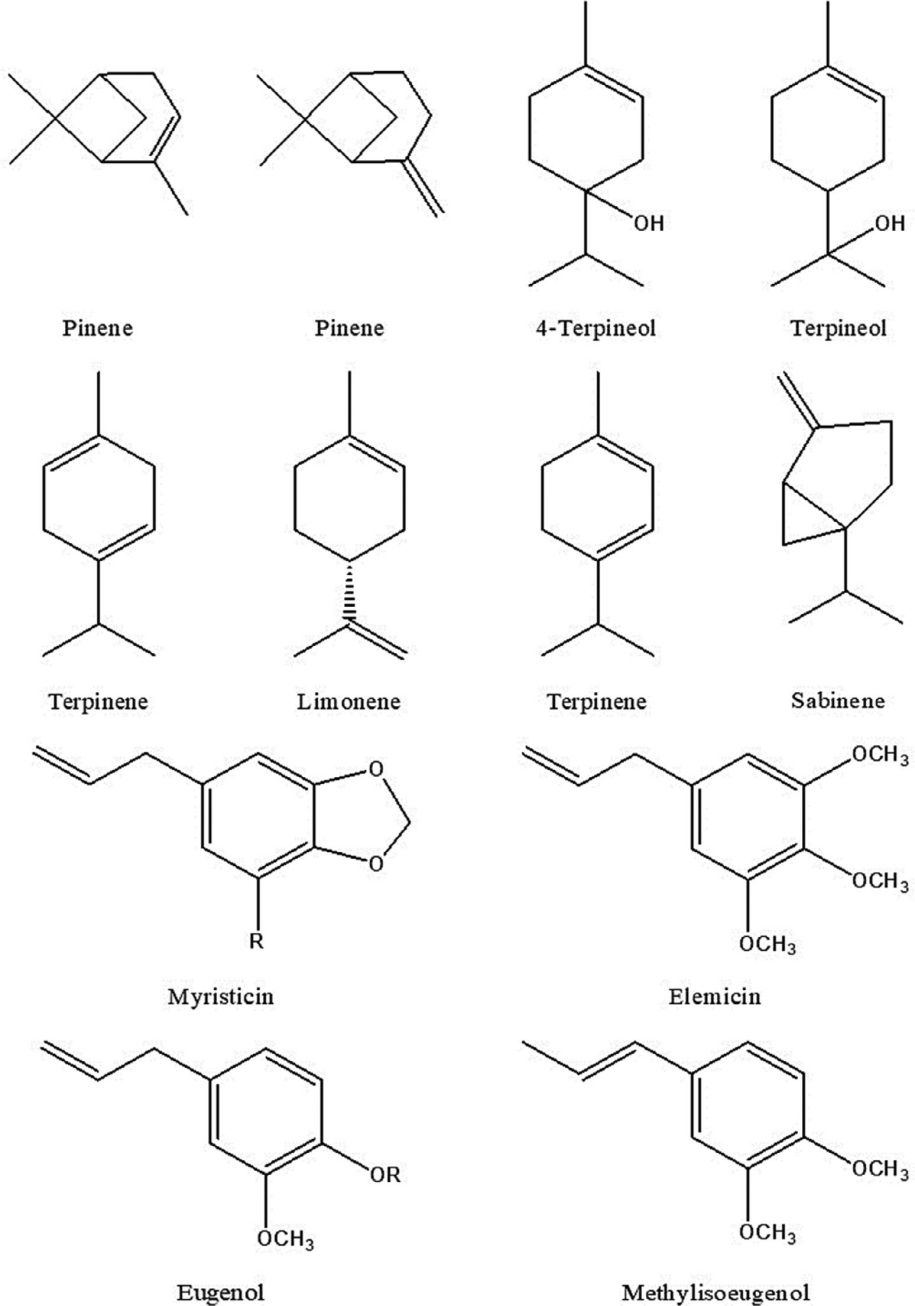
Some major compounds of *Myristica fragrans* Houtt. essential oils

Studies in recent research report showed that Western Ghats of South India grown nutmeg leaf oil (3.2% v/w) was found to have sabinene (17.2%), eugenol (16.6%), myristicin (9.1%), caryophyllene (8.8%), α‐pinene (5.4%), β‐pinene (6.4%), limonene (5.0%), β‐myrcene (4.7%), copaene (3.2%), germacrene D (3.0%), and 3‐Carene (2.7%), while mace oil (8.1% v/w) had sabinene (38.4%), α‐pinene (8.2%), β‐pinene (7.6%), limonene (7.1%), myristicin (5.9%), 3‐carene (5.1%), 4‐carene (4.2%), safrole (3.9%), β‐phellandrene (3.6%), and terpinen‐4‐ol (3.0%) as the major constituents (Ashokkumar et al., 2022). Furthermore, the Western Ghats (South India) grown nutmeg kernel (without rind/shell) are predominantly comprised of sabinene, α‐pinene, β‐pinene, limonene, and β‐myrcene (Table 2). However, these predominant chemical components were greater than those found in Pakistan grown nutmeg kernel oil (Atta‐ur‐Rahman et al., 2000). The higher concentration of oil components could be due to changes in soil type, location, season, and cultivars (Ashokkumar et al., 2021a; Ashokkumar et al., 2022). However, mace oil was rich in γ‐terpinene, safrole, terpinen‐4‐ol, α‐pinene, sabinene, and myristicin (Muhammad et al., 2016). Likewise, seeds from Pakistan contained EO consisting of sabinene, followed by α‐pinene, cymene, terpinen‐4‐ol, elemicin, and safrole (Table 2). Interestingly, the other nutmeg seed essential oil from Andaman & Nicobar Islands, India, extracted by hydrodistillation method, contained higher myristicin, sabinene, α‐thujene, α‐pinene, 4‐terpineol, limonene, γ‐terpinene, and elemicin (Waman, 2020). In a recent study, the Western Ghats (South India) grown nutmeg seed (with rind/shell) oil was predominantly composed of sabinene (27.7%) (Ashokkumar et al., 2022) and it was found to have a two‐fold lower concentration of sabinene (52.8%) in Grenada grown nutmeg seed EO (Mickus et al., 2021). The molecular structures of major EO constituents isolated from various parts of *M*. *fragrans* were drawn by ChemDraw software as shown in Figure 2.

**TABLE 2:** Major constituents of *Myristica fragrans* essential oils from various geographical origins

Geographical origins	Parts	Constituents	Reference
India (Western Ghats)	Leaf	Sabinene (17.2%), eugenol (16.6%), myristicin (9.1%), caryophyllene (8.8%), α‐pinene (5.4%), β‐pinene (6.4%), limonene (5.0%), β‐Myrcene (4.7%), copaene (3.2%), germacrene D (3.0%)	Ashokkumar et al. (2022)
India (Western Ghats)	Mace	Sabinene (38.4%), α‐pinene (8.2%), β‐pinene (7.6%), limonene (7.1%), myristicin (5.9%), 3‐carene (5.1%), 4‐carene (4.2%), safrole (3.9%), β‐phellandrene (3.6%), terpinen‐4‐ol (3.0%)	Ashokkumar et al. (2022)
Pakistan	Mace	γ‐Terpinene (19.1%), safrole (18.2%), terpinen‐4‐ol (12.7%), α‐pinene (11.6%), sabinene (11.2%), myristicin (7.5%)	Muhammad et al. (2016)
Sri Lanka	Kernel	Sabinene (43.4%), α‐pinene (17.5%), β‐pinene (12.1%), α‐phellandrene (4.3%), limonene (3.2%), terpinen‐4‐ol (3.5%), myristicin (3.0%)	Sarath‐Kumara, Jans, and Dharmadasa (1985)
India (Western Ghats)	Kernel	Sabinene (38.0%), α‐pinene (19.2%), β‐pinene (14.9%), limonene (7.2%), β‐myrcene (3.4%)	Ashokkumar et al. (2022)
India (Western Ghats)	Seed	Sabinene (27.7%), α‐pinene (21.8%), β‐pinene (18.2%), limonene (6.4%), β‐myrcene (2.9%)	Ashokkumar et al. (2022)
Indonesia (West Java)	Seed	Sabinene (21.4%), α‐pinene (10.2%), myristicin (10.6%), 4‐terpineol (13.9%), safrole (4.3%), γ‐terpinene (4.0%)	Muchtaridi et al. (2010)
India (Andaman & Nicobar Islands)	Seed	Myristicin (20.3%), sabinene (19.3%), α‐thujene (12.1%), α‐pinene (9.5%), 4‐terpineol (7.1%), limonene (5.9%), γ‐terpinene (4.1%), elemicin (4.0%)	Waman (2020)
Pakistan	Seed	Sabinene (18.9%), α‐pinene (15.8%), cymene (15.2%), terpinen‐4‐ol (11.7%), elemicin (11.5%), safrole (6.2%)	Muhammad et al. (2016)
Brazil	Seed	β‐Pinene (12.4–26.0%), sabinene (9.1–25.0%), α‐pinene (10.5–14.1%), myristicin (10.9%), γ‐terpinene (8.5%), limonene (6.3%), terpinen‐4‐ol (3.5%)	Valente, Jham, Dhingra, and Ghiviriga (2011); Cossetin et al. (2021)
Grenada	Seed	Sabinene (52.8%), α‐pinene (13.5%), α‐terpinyl acetate (6.0%), limonene (7.0%), γ‐terpinene (4.1%), β‐pinene (3.6%),	Mickus et al. (2021)

The yield of minor EO constituents of South India grown nutmeg mace are α‐phellandrene (1.9%), γ‐asarone (1.4%), p‐cymene (1.1%), α‐thujene (1.0%), methyleugenol (0.6%), caryophyllene (0.5%), α‐terpineol (0.4%), eugenol (0.4%), copaene (0.4%), β‐linalool (0.3%), germacrene‐D (0.3%), β‐terpineol (0.2%), and γ‐elemene (0.2%), while kernel oil had 3‐carene (1.8%), terpinen‐4‐ol (1.7%), copaene (1.7%), α‐thujene (1.5%), isoterpinolene (1.3%), γ–terpinene (1.2%), β‐terpineol (0.8%), γ‐asarone (0.8%), α‐phellandrene (0.8%), γ‐elemene (0.7%), myristicin (0.7%), p‐cymene (0.7%), 4‐carene (0.7%), caryophyllene (0.5%), p‐menth‐8‐en‐1‐ol (0.5%), safrole (0.5%), α‐terpinyl acetate (0.4%), β‐phellandrene (0.3%), camphene (0.3%), and α‐cubebene (0.3%) (Ashokkumar et al., 2022). Furthermore, the minor constituents of nutmeg seed essential oil are terpinen‐4‐ol (2.4%), copaene (2.4%), γ‐asarone (2.3%), β‐phellandrene (2.2%), myristicin (1.9%), α‐thujene (1.3%), 3‐carene (1.2%), β‐terpineol (1.1%), γ‐terpinene (1.0%), isoterpinolene (0.9%), p‐menth‐8‐en‐1‐ol (0.9%), γ‐elemene (0.8%), safrole (0.7%), methyleugenol (0.7%), 4‐carene (0.7%), p‐cymene (0.7%), α‐bergamotene (0.6%), α‐phellandrene (0.5%), camphene (0.4%), and α‐terpinyl acetate (0.4%) (Ashokkumar et al., 2022; Mickus et al., 2021; Waman, 2020). The variation in the minor components of MFEO might be due to the EO of four plant parts of nutmeg used.

## 4 BIOLOGICAL AND PHARMACOLOGICAL APPLICATIONS OF MFEO

**FIGURE 3:**
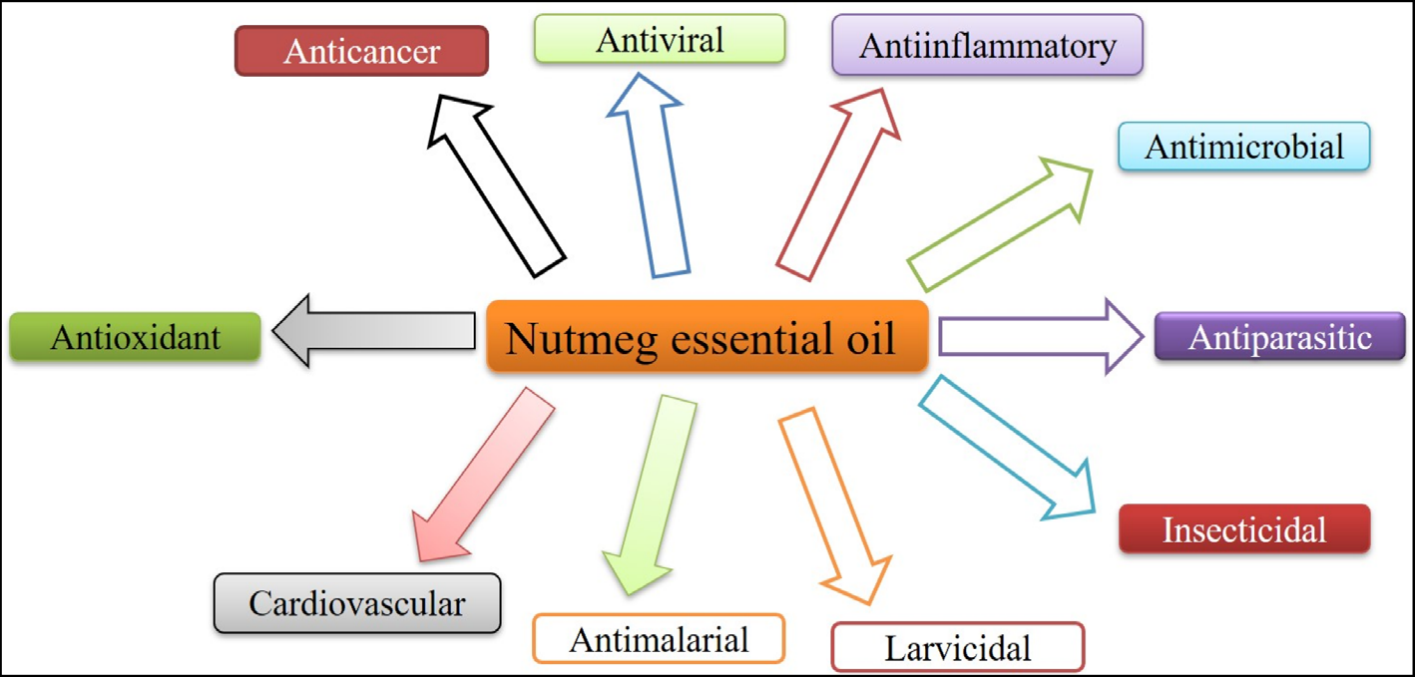
Diagrammatic representation of potential biological and pharmacological activities of *M*. *fragrans* essential oil

The pharmacological activities of *M*. *fragrans* extracts have been extensively reviewed (Asgarpanah & Kazemivash, 2012; Barceloux, 2009; Gupta, 2020; Jaiswal, Kumar, Singh, & Singh, 2009), which was not covered by pharmacological activities of MFEO. Therefore, we decided to review the pharmacological studies of MFEO and its active constituents up to 2022 that were not covered by previous reviews. The chemical constituents of various parts of MFEO are affected by several factors. However, different therapeutic applications such as antioxidant, antimicrobial, anticancer, and other miscellaneous activities are reported for MFEO and its active constituents in various literature. Furthermore, biological and pharmacological potential oil MFEO and its bioactive constituents were summarized in Tables 3 and 4, respectively. The potential biological and pharmacological activities of MFEO were diagrammatically presented in Figure 3.

**TABLE 3:** Biological activities of *Myristica fragrans* essential oil and its bioactive components

Biological activities	MFEO/active constituent	In vitro/in vivo	Target/ model	Control(s)	IC_50_/dosage	Results/remarks	Reference
Antioxidant activity	MFEO	In vitro	DPPH assay	Negative: Ethanol	EC_50_: 1.35 mg/ml	Good antioxidant activity observed after 20 min incubation	Nikolic et al. (2021)
Antioxidant activity	Elemicin 4‐terpineol Myristicin	In vitro	DPPH (Trolox equivalents) assay	Negative: Methanol	IC_50_:11.78 μM/g IC_50_:1.48 μM/g IC_50_:3.24 μM/g	The most potential antioxidant compound in the DPPH test was elemicin	Adiani, Gupta, Chatterjee, Variyar, and Sharma (2015)
Antioxidant activity	MFEO	In vitro	DPPH assay	Negative: Ethanol	0.2–20% concentration	Antioxidant activity was increased in a dose‐dependent manner.	Matulyte et al. (2020)
Antibacterial activity	MFEO	In vitro	*Pasteurella multocida*, *E*. *faecalis*, and *S*. *mutans*	Negative: DMSO	MIC: 0.2–1.0%	The concentration 0.2%, 0.5%, and 1% effectively suppress the growth of *P*. *multocida*, *E*. *faecalis*, and *S*. *mutans*, respectively.	Matulyte et al. (2020)
Antibacterial activity	MFEO	In vitro	*Escherichia coli* ATCC 25922	Positive: Gentamicin	MIC: 2.5%	MFEO have 10.89 mm inhibitory effects at 2.5%	Ansory, Fitriani, and Nilawati. (2020)
Antibacterial activity	MFEO	In vitro	*Bacillus cereus*, *L*. *Monocytogenes*, and *Micrococcus luteus*	Negative: DMSO	MIC: 83.3 μg/ml MIC: 79.2 μg/ml MIC: 88.1 μg/ml	The inhibition zone was 8.1, 9.0, and 8.2 mm, with the best inhibitory effect against *B*. *cereus*, *L*. *monocytogenes*, and *M*. *luteus*.	Purkait et al. (2018)
Antimicrobial activity	MFEO	In vitro	*B*. *subtilis*, *C*. *albicans*, *E*. *aerogenes*, *E*. *faecalis*, *E*. *durans*, *E*. *faecium*, *E*. *coli*, *K*. *pneumoniae*, and L. innocua,	–	MIC: 3.2 μg/ml −12.5 μg/ml	MFEO had displayed significant antimicrobial activity against all the microorganism.	Özkan et al. (2018)
Antibacterial activity	MFEO	In vitro	*S*. *aureus*, *B*. *subtilis*, *E*. *coli*, *S*. *typhi*, *K*. *pneumonia*, *P*. *aeruginosa*, and *B*. *pumilus*	Negative: Sterile water	MIC: 0.05% MBC: 0.1%	MFEO inhibited both Gram‐positive and Gram‐negative bacteria equally well	Cui et al. (2015)
Antifungal activity	MFEO	In vitro	*Aspergillus flavus* and *A*. *ochraceus*	Negative: Ethanol	MIC: 0.1% MIC: 0.3%	*A*. *flavus* and *A*. *ochraceus* growth were suppressed by 43 and 65%, respectively, at a concentration of 0.1% of MFEO. 0.3% inhibited *A*. *flavus* and *A*. *ochraceus* growth by 84 and 79%, correspondingly	Valente et al. (2015)
Antifungal activity	MFEO	In vitro	*Candida tropicalis* ATCC 13803, *C*. *krusei* ATCC 6258, *C*. *albicans* ATCC 90028, *C*. *glabrata* ATCC 90030, *C*. *parapsilosis* ATCC 22019, *C*. *albicans*	Negative: Sterile water	MIC: 0.31–2.5 μg/ml MBC: 0.31–2.5 μg/ml	Inhibition zone ranged between 8.3 and 30 mm with anticandidal activity against all tested *Candida* sp.	Thileepan et al. (2018)
Antimicrobial activity	MFEO	In vitro	*C*. *albicans* ATCC2091, *S*. *aureus* ATCC 25923, *B*. *cereus* ATCC 11778, *B*. *luteus*, *E*. *coli* ATCC 25922, *K*. *pneumoniae* ATCC 700603	Positive: Nystatin	MIC: 0.1%	The inhibition zone was 28, 14, 14, 23, 14, and 15 mm with the best inhibitory effect against *C*. *albicans*, *S*. *aureus*, *B*. *cereus*, *B*. *luteus*, *E*. *coli*, and *K*. *pneumoniae*, respectively	Nikolic et al. (2021)
Antimalarial activity	MFEO	In vitro	*Plasmodium falciparum* D6	–	MIC: 16 μg/ml	MFEO showed some antimicrobial activities	Ibrahim et al. (2020)
Fumigant activity	MFEO	In vivo	*Callosobruchus maculatus*	Negative: Untreated beans	LC_50_: 30 μl/L for 24 hr	100% mortality of adults *C*. *maculatus* were observed	Alibabaie and Safaralizadeh (2015)
Larvicidal activity	MFEO	In vivo	*Aedes aegypti*	Negative: Distilled water	LC_50_: 110.1 μg/ml for 24 hr	Significant larvicidal activity was noticed	Carolina and Maman (2016)
Insecticidal activity	MFEO	In vivo	*Lasioderma serricorne*	Negative: Hexane	LD_50_: 19.3 mg/adult for 24 hr	MFEO exhibited strong contact toxicity against *L*. *serricorne*	Shu‐Shan et al. (2014)
Insecticidal activity	Elemicin	In vivo	*Lasioderma serricorne*	Negative: Hexane	LD_50_: 9.8 mg/adult for 24 hr	Elemicin showed strong contact toxicity against *L*. *serricorne*	Shu‐Shan et al. (2014)
Insecticidal activity	MFEO	In vivo	*Chrysomya albiceps* and *Musca domestica*	–	LC_50_: 2.2 μg/ml LC_50_: 8.6 μg/ml	Topical application of MFEO was toxic to *C*. *albiceps* and *M*. *domestica*	Cossetin et al. (2021)

*Note*: Not reported; EC₅₀, Half maximal effective concentration; IC₅₀, Half maximal inhibitory concentration; MFEO, *Myristica fragrans* essential oil; LC_50_, Lethal concentration 50%; LD_50_, Lethal dose 50%; MIC, Minimum inhibition concentration; MBC, Minimum bactericide concentration.

**TABLE 4:** Pharmacological activities of *Myristica fragrans* essential oil and its active components

Biological activities	MFEO/active constituent	In vitro/in vivo	Target/model	Control(s)	IC, LD_50_/dosage	Results/remarks	Reference
Cytotoxic activity	MFEO	In vitro	Vero cell line	–	IC_50_: 24.8 μg/ml	Low cytotoxicity observed against Vero cell line	Piaru et al. (2012b)
Anticancer activity	MFEO	In vitro	Human colon adenocarcinoma cell line (undifferentiated Caco‐2 cells)	Positive: Myristicin	IC_50_: 250 μg/ml	MFEO were found to have a considerable inhibitory effect on the growth of a colon cancer cell line	Piras et al. (2012)
Antiinflammatory activity	MFEO	In vitro	hTERT‐immortalized foreskin fibroblast cell line, BJ‐5ta treated with viral dsRNR mimetic poly I:C	Negative: 96% ethanol	LD_50_: 1 mg/ml	Nutmeg essential oils and hydrolats have shown significant antiinflammatory effect protecting cell viability	Matulyte et al. (2020)
Hepatoprotective activity	MFEO	In vivo	Male albino mice	–	500, 1,000, mg/kg for 24 hr	MFEO‐treated mice showed substantial alteration in the biochemical indicators of liver function in a dose‐dependent manner	Al‐Jumaily & Al‐Amiry, 2012
Antiinflammatory activity	MFEO	In vivo	Complete Freund's adjuvant (CFA)‐injected rats (30 mg/kg/day)	Positive: Diclofenac	20 mg/kg/day	MFEO potentially alleviated the CFA injection‐induced joint swelling, mechanical allodynia of rats through inhibition of COX‐2 expression, and blood substance P level	Zhang et al. (2016)
Anticonvulsant activity	MFEO	In vivo	Male mice	Positive: Valproic acid	LD_50_:2150 μL/kg for 24 hr	Substantial anticonvulsant activity was observed	Wahab, Ul Haq, Ahmed, Khan, and Raza (2009)
Anticonvulsant activity	α‐Terpineol	In vivo	Genetic absence epilepsy rats from Strasbourg (GAERS) rats	Positive: Diazepam	10, 20, 50 mg/kg, i.p.	Intraperitoneal dose 10 mg/kg was less effective to control seizures and spike and wave discharges (SWDs) in GAERS rats, but 20 mg/ kg and 50 mg/kg (i.p.) decreased the number of seizure episodes and number of SWDs	Islam et al. (2012)

*Note*: Not reported; IC_50_, inhibitory concentration, LD_50_, lethal concentration; MFEO, *Myristica fragrans* essential oil.

### 4.1 Antioxidant activities

The antioxidant activity of MFEO can be dignified through testing chemical assays like DPPH (2,2‐diphenyl‐1‐picrylhydrazyl), ferric reducing/antioxidant power assay (FRAP), inhibition of lipid peroxidation, and bleaching of β‐carotene (Gupta, Bansal, Babu, & Maithil, 2013). The nutmeg essential oil showed 88.7% inhibition in linoleic acid oxidation with an EC_50_ dosage of 181.4 μg/ml (Piaru et al., 2012a). Matulyte et al. (2020) noticed that pure MFEO and MFEO with 1% of magnesium aluminometasilicate had similar antioxidant activity for EO concentrations of 0.2–20%. Also, this study noted that antioxidant activity was increased in a dose‐dependent manner.

In another study, Adiani et al. (2015) assessed the antioxidant potential of MFEO bioactive constituents like elemicin, myristicin, 4‐terpineol, and sabinene, and among them, elemicin was an effective antioxidative agent confirmed by DPPH assay. In a recent study, MFEO with an EC_50_ dosage of 1.35 mg/ml showed significant antioxidant activity after 20 min of incubation (Nikolic et al., 2021). However, most studies of antioxidant activity based on chemical testing, such as DPHH assays, are no longer relevant to pharmacology. For these effects to be regarded as clinically relevant, their potential under in vitro conditions must be investigated (Harnly, 2017).

### 4.2 Antimicrobial activity

Nowadays, the utilization of EO and its natural compounds for controlling food‐borne bacteria and fungi microorganisms in food and by‐products have increased the demand for food industries due to its green policies. The essential oils of spices have been reported to inhibit microorganism growth (Ashokkumar et al., 2020a, 2020b, 2021b) and are insoluble in water. To date, only a few studies were conducted to investigate the antimicrobial activity of MFEO. The MFEO with 0.5% concentration was sufficient to complete suppression in the growth of *E*. *faecalis*, and *S*. *mutans* even 0.2% concentration effective to suppression of *Pasteurella multocida* growth. Thus, this study results also suggested that the combination of MFEO and magnesium aluminometasilicate (1%) widens the spectrum of antimicrobial activity (Matulyte et al., 2020). According to Soni, Sharma, and Jasuja (2016), dilution of MFEO from 8 to 14 μl (25% v, v) had significant antibacterial activity against Gram‐negative bacterial strains *E*. *coli*, *P*. *vulgaris*, and *K*. *pneumoniae* and Gram‐positive bacterial strains like *S*. *aureus*, and *B*. *subtilis* using agar well‐diffusion method. Özkan et al. (2018) also found that the EO of nutmeg has potent antibacterial activity against *S*. *typhimurium* with MIC value of 1.6 μg/ml.

Nurjanah, Putri, and Sugiarti (2017) examined Indonesia grown nutmeg seed oil for antibacterial activity by disc diffusion method using different concentrations of MFEO, 20, 40, 60, 80, and 100%, and highest inhibition zone was observed on 60% concentration for *S*. *aureus*, *S*. *epidermis*, *S*. *dysenteriae*, and *S*. *typhi*. The Brazilian nutmeg EO (MIC: 0.1%) had inhibited the growth of *Colletotrichum gloeosporioides* (98%), *C*. *musa* (97%), *Fusarium oxysporum* (75%), *F*. *semitectum* (78%), *Aspergillus niger* (71%), and *A*. *glaucus* (60%). Also, 0.3% concentration of MFEO increased the inhibition growth from 85% to 100% (Valente et al., 2011). In a recent study, Ansory et al. (2020) observed that Indonesian nutmeg EO, MIC: 1.2% and MBC: 2.5%, substantially inhibited *Shigella* sp. growth. The majority of studies focusing on the antimicrobial activity of MFEO have been conducted using the disc diffusion method. However, because of its flaws, the method needs to be paired with the more relevant MIC assay (Van Vuuren, 2008).

### 4.3 Antiinflammatory and analgesic activity

Inflammation plays an imperative role in the human body. The antiinflammatory and analgesic effects of MFEO were investigated using rats injected with Complete Freund's adjuvant (30 mg/kg/day). Daily intraperitoneal injections of nutmeg oil [CFA + Nitric oxide (NO) high, 20 mg/kg/day and CFA + NO low, 10 mg/kg/day] may reduce CFA injection caused by joint swelling, mechanical allodynia, and heat hyperanalgesia in rats by inhibiting COX‐2 expression and blood substance P levels (Zhang et al., 2016). In a recent study, Matulyte et al. (2020) investigated the antiinflammatory activity of MFEO and hydrolats in the hTERT‐immortalized foreskin fibroblast cell line, BJ‐5ta, treated with viral dsRNR mimetic Poly I: C. The study results reported that both the MFEO and hydrolats had shown significant antiinflammatory effects. Only a few studies have looked into MFEO's antiinflammatory and analgesic properties, and they were all done in animal models rather than humans. Future research should look into the bioactivity of MFEO in different clinical studies with people.

### 4.4 Insecticidal and nematicide activities

Gotke, Maheshwari, and Mathur (1990) noticed that the mace oil effectively controlled root‐knot nematode (*Meloidogyne incognita*) than nutmeg kernel oil. MFEO substantially inhibits *T*. *castaneum* eggs hatching and following survival of larvae in the concentration range 1.4–3.2 mg/cm^2^. The production of F_1_ progeny was completely suppressed at MFEO concentration of 0.35 g/100 g wheat for *Sitophilus zeamais*, and 1.05 g/100 g rice for *Tribolium castaneum*. The feeding deterrence index 7% and 33% was observed for *Tribolium castaneum* and *Sitophilus zeamais*, respectively, at a concentration of 20 g nutmeg oil/100 ml (Huang, Tan, Kini, & Ho, 1997). The concentration of 10 mg/ml of nutmeg EO revealed highest fumigant action and contact toxicity against whitefly adults and whitefly nymphs, respectively (Wagan, Wang, Hua, & Cai, 2017). MFEO also had significant antifeedant activity against larvae of *Lymantria dispar* (Kostic et al., 2013). The oviposition of cowpea storage bruchid (*Callosobruchus maculates*) was effectively inhibited by nutmeg oil, and 60% mortality and 85% mortality were observed at 2% dosage on the 3rd day after application and 7‐day post‐treatment, respectively. Another study also reported that MFEO inhibited adult emergence of *C*. *maculates* (Adedire, 2002). In a recent study, adult house fly (*Musca domestica*) and *Chrysomya albiceps* were subjected to MFEO impregnated paper with LC_50_ values of 2.74 g/ml and 3.65 g/ml, respectively. As a result, the findings demonstrated that MFEO has insecticidal activity and can be utilized to control *M*. *domestica* and *C*. *albiceps* (Cossetin et al., 2021). So far, only a few studies have investigated the insecticidal properties of MFEO, so more research is needed in this promising biological field.

### 4.5 Miscellaneous activities

Myristicin is one of the vital chemical compounds of MFEO. Ingestion of myristicin attributed various antagonistic effects, including vomiting, gastrointestinal, tiredness, cardiovascular, and hypotension (Grover, Khandkar, Vats, Dhunnoo, & Das, 2002). In mice, myristicin‐suppressed lipopolysaccharide/d‐galactosamine (LPS/D‐GalN) induced increases in serum TNF and hepatic DNA fragmentation, indicating that it possesses significant hepatoprotective effect (Morita et al., 2003). Besides, myristicin had potential apoptotic, anthelmintic, and insecticidal activities (Lopez et al., 2015; Martins et al., 2014). Furthermore, most of these effects are limited in the literature, with no logical follow‐up investigations that further validate outcomes. MFEO showed antiamoebic activity against *Entamoeba hystolytica* (De Blasi, Debrot, Menoud, Gendre, & Schowing, 1990). In another study, MFEO showed significant antiangiogenic activity with IC_50_ of 77.6 μg/ml compared with *Morida citrifolia* oil which exhibits IC_50_ of 109.3 μg/ml (Piaru et al., 2012a). At a concentration of 200 g/ml, MFEO has significant antiangiogenic properties. Antiangiogenesis is a process that inhibits the creation of new blood vessels in tumors in order to slow their growth (Al‐Rawi et al., 2011). Kholibrina and Aswandi (2021) reported that the combined EOs of nutmeg, citronella, and benzoin significantly reduced hypertension. However, this study lacks systematic experiment protocols like positive and negative control and dosages. Therefore, future studies need to conduct the experiment as systematically as possible to confirm the hypertensive activity of the MFEO. Furthermore, some additional pharmacological potential of MFEO, and their active constituents was also summarized in Table 4.

## 5 CONCLUSION AND FUTURE OUTLOOKS

In this review, we summarize the knowledge of botanical description, oil yield, chemical composition and biological activities of *M*. *fragrans* EO. The plant essential oil yield and chemical compositions focused on mace, leaves, and kernels, but barks and roots have been neglected or have received less attention. Among the various essential oil extraction methods, hydrodistillation is predominantly used for essential oil extraction. Chromatographic techniques are used for the identification of chemical constituents from essential oil. The primary chemical constituents of MFEO were sabinene, eugenol, myristicin, caryophyllene, β‐myrcene, α‐pinene, β‐pinene, D‐limonene, and 3‐carene. These predominant chemical constituents of nutmeg can serve as a novel potential natural source, which can be used for food, perfumery, and pharmaceutical industries. Our literature survey found that MFEO can protect people from several diseases due to their potent biological activities. Among the previous biological and pharmacological studies, most of them did not report positive and negative controls details (Tables 3 and 4). Hence, future studies need to conduct systematic studies using cell and animal models. Clinical and experimental investigations have confirmed the antioxidant, antibacterial, antimalarial, antifungal, anticonvulsant, antiinflammatory, analgesic, anticancer, apoptotic, anthelmintic, antiangiogenic, antiamoebic, and insecticidal activities of MFEO. However, these clinical studies were conducted only in animals and cell lines, and no human clinical trials have been conducted. As a result, future research should concentrate on MFEO's and their active constituents' pharmacological activities in humans. Future research should look at the toxicity, bioavailability, and pharmacokinetics of MFEO to find the chemical components responsible for its activities and expand the existing medical application of *M*. *fragrans*. We believe that the information provided or discussed here will increase public awareness of MFEO and be valuable for future research.

## AUTHOR CONTRIBUTIONS

Kaliyaperumal Ashokkumar, Jesus Simal‐Gandara, and Muthusamy Murugan conceived and designed the review. Kaliyaperumal Ashokkumar and Jesus Simal‐Gandara wrote the manuscript. Mannananil Krishnankutty Dhanya and Arjun Pandian collected literature and drew molecular structures of major nutmeg essential oil compounds by ChemDraw software. Jesus Simal‐Gandara and Muthusamy Murugan edited the manuscript. All the authors reviewed and approved this version of the manuscript.

## FUNDING INFORMATION

Funding for open access charge: Universidade de Vigo/CISUG.

## CONFLICT OF INTEREST

The authors declare no conflict of interest in this article.

## Data Availability

Data sharing is not applicable to this article as no new data were created or analyzed in this study.
